# Development and questionnaire-based evaluation of virtual dental clinic: a serious game for training dental students

**DOI:** 10.1080/10872981.2021.1983927

**Published:** 2021-10-25

**Authors:** Ju-Hui Wu, Je-Kang Du, Chen-Yi Lee

**Affiliations:** a Department of Oral Hygiene, College of Dental Medicine, Kaohsiung Medical University, Kaohsiung, Taiwan; b Department of Dentistry, Kaohsiung Medical University Hospital, Kaohsiung, Taiwan; c School of Dentistry, College of Dental Medicine, Kaohsiung Medical University, Kaohsiung, Taiwan; d Department of Medical Research, Kaohsiung Medical University Hospital, Kaohsiung, Taiwan

**Keywords:** Clerkship, dental education, family dentistry, serious game, virtual dental clinic

## Abstract

**Background:**

The volume of literature about serious gaming in dental education has increased, however, none of the previous studies have developed a serious game for closing the gap between preclinical and clinical training.

**Objective:**

Virtual Dental Clinic (VDC) is a serious game that was created to help develop clinical reasoning skills in dental students. This study aimed to evaluate VDC as an educational tool and its effectiveness on clinical skill and knowledge gain among clerkship dental students.

**Methods:**

The following three stages of VDC design and testing were addressed from 2016 to 2020: development, validation, and application. The VDC was developed using Unity game engine. In the validation stage, the content validity was reviewed by five visiting staff; construct validity and face validity were examined by 9 postgraduate-year dentists and 14 clerkship dental students. Concurrent validity and predictive validity were examined by 34 fifth-year dental students during their clerkship from September, 2018 to May, 2019, the associations between VDC experiences, clerkship performance, and the score on a national qualification test were explored. In the application stage, the VDC was set up as a self-learning tool in the Family Dentistry Department from August, 2019, quantitative and qualitative analyses were conducted using the 92 clerkship students’ feedback.

**Results:**

The VDC showed good validity and a high potential for education in practice. Students who have used VDC received significantly higher scores on qualification test (p = 0.029); the VDC experiences significantly predicted higher performance score on periodontics (p = 0.037) and endodontics (p = 0.040). After the outbreak of COVID-19 pandemic, significantly higher proportion of students confirmed the value of VDC as an assistant tool for learning clinical reasoning (p = 0.019).

**Conclusions:**

The VDC as an educational tool, and the effectiveness on clinical reasoning skills and knowledge gain among clerkship dental students has been validated and confirmed in this study.

## Introduction

Dental education is usually divided into two parts (preclinical and clinical). Preclinical students predominantly study basic science in lectures and practical work in the simulation laboratory courses. In the clinical part, students work with actual patients under the supervision of visiting staff [1]. Transition points in dental education can be abrupt, stressful, and disorienting for learners. The first transition point for students, after dental school matriculation, is the move from the preclinical curriculum to clinical clerkships. Closing the gap between preclinical and clinical training is essential [2]. Preclinical dentistry training is the preparatory phase that includes integrated basic science knowledge and skills in a decision-making context. This is expected to enable future clinicians to independently manage diverse clinical scenarios and deliver adequate high-quality care to patients [3,4]. Accordingly, training programmes should provide students a safe environment that is conducive for learning anywhere and anytime.

Serious games are technology-enhanced simulation games developed for a purpose other than entertainment, such as teaching a specific knowledge or skill [5]. Unlike traditional approaches, they are often considered to provide a more interactive learning environment, a motivational approach to learning, and safe training for patients and students. They also allow students to reconsider their strategies following failure until they complete the game [6,7]. Positive attitude and learner satisfaction have been found in medical, health professional, and dental students using these games as a learning tool [8,9]. However, the application of serious games in dental education remains underutilised and under-researched [9].

Previous studies related to simulation in dental education mainly focused on the simulated training on psychomotor skills [10]. Serious game for improving the transition of declarative knowledge to procedural knowledge is a new attempt. Two studies [11,12] used randomised control trials to make comparisons between serious games (test group) and traditional learning style (control group) in dental education. Amer [11] reported on using a serious game for teaching dentine bonding and basic knowledge for composite resin filling in operative dentistry. Hannig et al. [12] assessed the effectiveness of Skills-O-Mat, a serious game for training in mixing alginate for dental impression, which is a mandatory skill for dental care. Overall, two key studies [11,12] in our knowledge, suggest that serious games are potentially effective learning tools for dental education, and the students were satisfied with the game-based learning approach. Both studies [11,12] were performed on first-year or second-year dental students to acquire basic science and skills. Games Research Applied to Public Health with Innovative Collaboration-II (GRAPHIC-II) [13] is a serious game for dental public health where students can practice critical thinking and decision-making skills regarding health promotion in a safe environment.

In Taiwan, treatment planning is one of the most important nonoperational skills for dental students [14]. However, to the best of our knowledge, previous studies have not examined the effectiveness of a serious game in helping students with treatment planning and familiarising them with the procedures of various treatments. Virtual Dental Clinic (VDC) is a serious game developed for training clinical reasoning in dentistry. Clinical reasoning is a vital competency that enables students to apply the theory they learned to actual clinical situations. This study aimed to evaluate the validity and effectiveness of VDC as an educational tool for clinical skills and knowledge gain among clerkship dental students.

## Materials and methods

**Figure 1.**
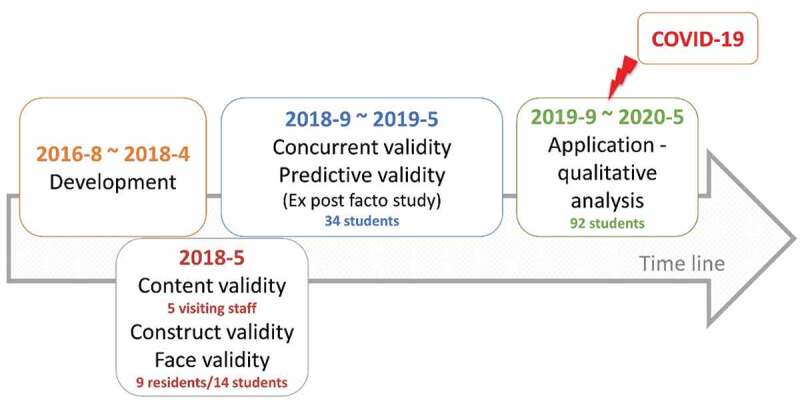
The development and validation of the virtual dental clinic

The following three stages of VDC design and testing were addressed. The sequence involved development, validation, and application (Figure 1). In the development stage, from August 2016 to April 2018, the prototype of a dental simulation clinic software was prepared, designed, and developed. In the validation stage, during May 2018, 5 visiting staff were recruited to examine the content validity of each theme, 9 residents and 14 students were recruited to examine the responses of the experts and novices (construct validity and face validity); from September 2018 to May 2019, a quasi-experimental ex post facto study was conducted on a convenience sample of 34 fifth-year students during their clerkship training, the concurrent validity and predictive validity were obtained by analysing their academic achievements. Finally, in the application stage, the VDC was introduced to the new batch of fifth-year students as a self-learning tool during their clerkship training, from September 2019 to May 2020, and a qualitative analysis was conducted. The study design followed the suggested framework for game development [15] and criteria for the validation process [16–18].

### Ethics

This study was approved by the Institutional Review Board of Kaohsiung Medical University Hospital (Letter No. KMUHIRB-SV(II)-20170009 and KMUHIRB-SV(II)-20210020). Written informed consent was obtained from all participants.

### Description of the serious game and its development

**Figure 2.**
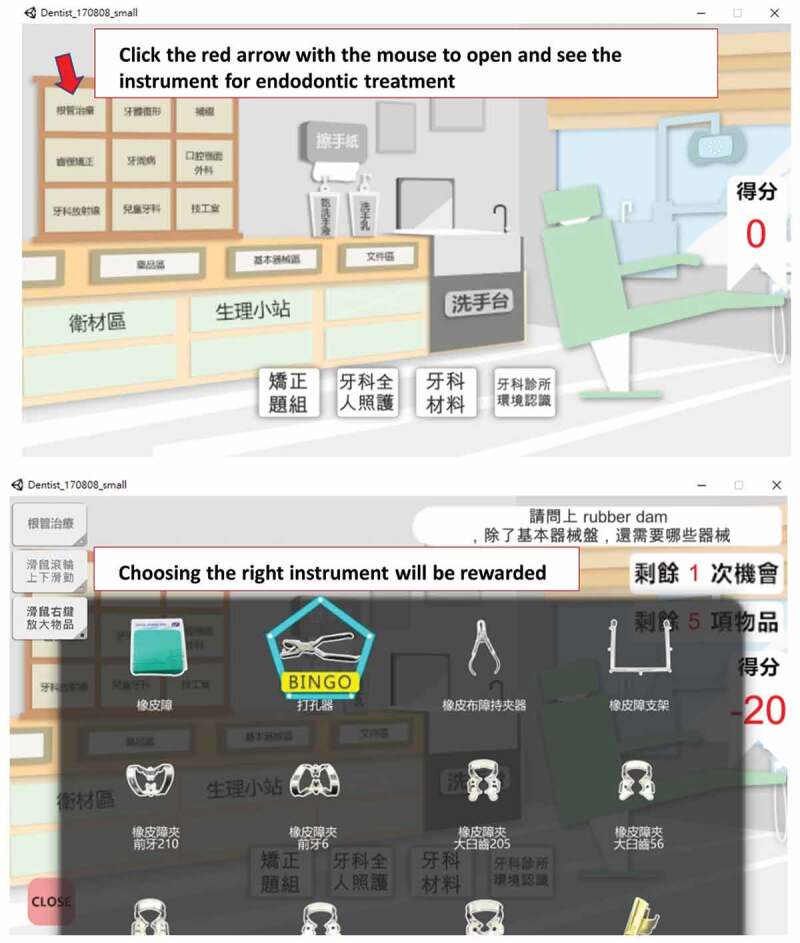
Screenshots from the virtual dental clinic

The concept of this dental simulation clinic software was originally designed by Ju-Hui Wu (one of the authors of this paper). This gaming software is developed using the Unity game engine and built on a stand-alone game running on the Windows platform. The learning objective is to improve the cognitive knowledge of dental clerks regarding various treatment procedures, such as tooth composite resin filling, root canal therapy, and cement mixing methods, and also enhance the understanding of materials and equipment in the clinic, to know the timing of their use and operation methods. The game scene is a virtual dental clinic, including the dental equipment, materials, documents, and medicines built in the game software (Figure 2). The player is required to click the red arrow with the mouse to open and see the instrument for endodontic treatment. Choosing the right instrument is rewarded. The task is divided into six themes: introduction of dental clinic environment, interpretation of X-ray images, dental materials, orthodontics instrument, patient care in operative dentistry, and endodontic treatment. Each theme is subdivided into 2–12 items, with 42 items in total. During the game, a failure message appears when the answer is wrong, and an applause when the answer is correct. The game provides immediate feedback to the player and using a formative assessment method. The player has two chances to answer a question.

### Validation of the serious game

#### Content validity

Five visiting staff with at least three years of experience in general dentistry were invited to review the items of each theme. The appropriateness of each item was rated on a 5-point Likert scale (ranging from 1 = not relevant to 5 = highly relevant). Next, for each item, the item-level content validity index (I-CVI) was calculated based on the number of experts who rated it as either 4 or 5 (thus, dichotomising the ordinal scale into *relevant* and *not relevant*) divided by the total number of experts. An item that was rated as *relevant* by four out of the five experts would have an I-CVI of 0.80 [19,20]. They suggested that six items needed modification. After the modification, all items showed an acceptable item-level content validity index (I-CVI ranged from 0.8 to 1) (see Appendix).

There are two methods to calculate the scale-level content validity index (S-CVI) – using the universal agreement calculation methods (S-CVI/UA) and estimating by averaging calculation methods (S-CVI/Ave) [19,20]. In this study, both ways of calculating the S-CVI were found to be acceptable (S-CVI/UA = 0.81, S-CVI/Ave = 0.96) (see Appendix). The S-CVI/Ave of each theme is reported according to the recommendations of Polit et al. [20].

#### Construct validity and face validity

A total of 9 postgraduate-year (PGY) dentists and 14 clerkship dental students who were training in Family Dentistry Department at that time were invited to play the VDC, and their answers in the first round for each item of all themes were recorded. We then compared the rates of correct answers between experts and novices.

After finishing the game, they were asked to fill a questionnaire, which included their impressions and attitudes toward VDC’s educational values and game quality. The content of the questionnaire was modified from Wang et al. [21], including eight items for educational values and seven items for game quality, measured on a 5-point Likert scale (1 = strongly disagree to 5 = strongly agree). The Cronbach’s alpha coefficients of 0.908 for educational value and 0.905 for game quality showed good internal consistency.

#### Concurrent validity and predictive validity

A quasi-experimental ex post facto study was conducted. A total of 34 fifth-year dental students volunteered to participate in this study before they began the clerkship from September 2018 to May 2019. The participants were given the VDC software, and informed that they have the right decide if they wished to use the VDC as a self-learning tool during their clerkship or not. Those who had used VDC were categorised as experimental group and those who had not were categorised as the control group. Information pertaining to their video game-playing habits and learning strategies were collected.

**Figure 3.**
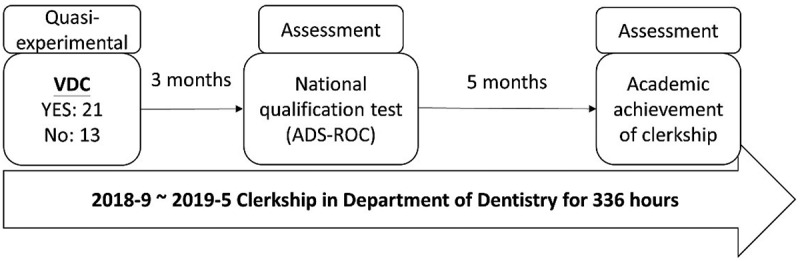
The timeline of VDC self-learning and related assessments

In their clerkship training, they were required to complete 84 sessions, involving 336 hours of training in the department of dentistry at a medical centre. Before they finished the training, all the fifth-year dental students were required to attend a national qualification test hosted by the Association for Dental Sciences of the Republic of China (ADS-ROC). This test which was divided into two parts – the operational test and the computer-based test for non-operational skills – aimed to evaluate each student’s preparedness to be an intern. The participants’ computer-based test scores were used to evaluate the VDC’s concurrent validity. The computer-based test comprised four clinical cases and aimed to exam the abilities related to clinical reasoning, treatment planning, and instrument selection. After finishing the clerkship training, the participants’ academic achievements were evaluated by the tutors of each specialty. We used the performance scores to evaluate the predictive validity of VDC (Figure 3).

### Practical application

From August, 2019, the Family Dentistry Department set up the self-learning tools including the textbooks, simulation model, and VDC for students to create a self-learning environment. There were 92 fifth-year dental students who volunteered to use these self-learning tools from September, 2019 to May, 2020 during their clerkship training. Although COVID-19 occurred during this period, Taiwan implemented an effective epidemic control policy, so students’ clerkship training was unaffected. Thus, the practical application of VDC was also unaffected and could be analysed in this study. In fact, due to COVID-19, self-learning, and hence, effective self-learning tools, have become essential for teaching-learning processes. Once the clinical training course finished, the students were required to complete some homework regarding their training at the Family Dentistry Department. This homework was included in the qualitative analysis.

### Statistical analysis

All data were analysed using IBM Statistical Package for Social Sciences (SPSS) for Windows version 20.0. For all statistical tests, a significance level of p < 0.05 was used. Regarding the validation process, details pertaining to the content validity are provided in the Appendix. To compare the difference between the responses of experts and novices to each item, the Likert scale was changed to agree (4 ≥) and disagree (≤ 3) for the Fisher’s exact test, to compare the differences between rates of correct scores of experts and novices for each theme, the impressions of VDC, and their evaluation of game quality and usability for education (construct validity and face validity). We used independent sample *t*-test and one-way analysis of variance (ANOVA) to analyse the influence of game-playing habit, learning strategies, and VDC experiences on qualification test and performance scores of each department (concurrent validity and predictive validity). Regarding the application stage, we again used Fisher’s exact test to compare the extents of self-learning and VDC recommendation of clerkship students before and after COVID-19 epidemic. A qualitative analysis was conducted to summarise the recommendations of the students.

## Results

### Content validity of the themes

The original S-CVI/Ave of themes ranged from 0.73 to 1.0. After modifying six items, the S-CVI/Ave of all the themes improved, as shown in Table 1.

**Table 1.** The S-CVI of VDC

Themes		Items	S-CVI/Ave	
			Original	Modified
1	Dental clinic environment	10	0.90	1.00
2	Dental materials	2	0.90	n/a
3	Whole-person dental care: first visit (OD)	6	0.73	0.90
4	Whole-person dental care: second visit (ENDO)	6	0.80	0.87
5	X-ray arrangement	10	0.98	n/a
6	Orthodontics	8	1.00	n/a
Total		42	0.90	0.96

### Correct answer rate comparison between experts and novices

Table 2 shows the comparison of correct answer rates when playing VDC between PGY dentists and clerkship dental students. The PGY dentists achieved 100.0% correct rate on all themes. The easiest theme for clerkship students was ‘X-ray arrangement’ (100.0% correctness), followed by ‘dental clinic environment’ (78.6%). The themes regarding ‘whole-person care’ and ‘orthodontics’ were difficult for them. The comparison revealed significant differences between experts and novices.

**Table 2.** The proportions of full score between expert and novice

Themes		Full score, n (%)		p	Difficulty level
		Clerk (n = 14)	PGY (n = 9)		
1	Dental clinic environment	11 (78.6)	9 (100.0)	0.136	Easy
2	Dental materials	8 (57.1)	9 (100.0)	0.022*	Moderate
3	Whole-person dental care: first visit (OD)	1 (7.1)	9 (100.0)	0.000***	Difficult
4	Whole-person dental care: second visit (ENDO)	0 (0.0)	9 (100.0)	0.000***	Difficult
5	X-ray arrangement	14 (100.0)	9 (100.0)	n/a	Easy
6	Orthodontics	1 (7.1)	9 (100.0)	0.000***	Difficult

Fisher’s exact test.

* p < 0.05; *** p < 0.001.

### Impressions of VDC

The clerkship students reported that the most positive impression of VDC was ‘effective in education’ (92.9%), followed by ‘teach dental knowledge’ (85.7%); the PGY dentists reported that the most positive impression was ‘teach clinical skills’ (100.0%). Overall, the clerkship students and PGY dentists had a positive attitude toward VDC’s educational values and showed no significant differences for any item (Table 3).

**Table 3.** The impressions of VDC

Items		Agree, n (%)			p
		Total	Clerk (n = 14)	PGY (n = 9)	
1	Effective in education	19(82.6)	13 (92.9)	6 (66.7)	0.147
2	Teach dental knowledge	18 (78.3)	12 (85.7)	6 (66.7)	0.283
3	Teach clinical skills	20 (87.0)	11 (78.6)	9 (100.0)	0.206
4	Effective feedback	16 (69.6)	9 (64.3)	7 (77.8)	0.418
5	Sense of presence and immersive	18 (78.3)	11 (78.6)	7 (77.8)	0.673
6	Fun, wants to play again	19 (82.6)	11 (78.6)	8 (88.9)	0.483
7	Long-lasting learning efficacy	16 (69.6)	10 (71.4)	6 (66.7)	0.582
8	Necessary for learning	15 (65.2)	8 (57.1)	7 (77.8)	0.290

Fisher’s exact test.

### Game quality and usability for education

Table 4 shows the game quality and usability as evaluated by clerkship students and PGY dentists. The most positive evaluation was for ‘the test design is good for learning’ from the clerkship (85.7%) and ‘it’s good for preview’ (88.9%) from the PGY dentists. Overall, the clerkship students and PGY dentists had a positive attitude toward VDC’s game quality and usability. There were no significant differences between the responses of the two groups for any item.

**Table 4.** Game quality and usability of VDC

Items		Agree, n (%)			p
		Total	Clerk (n = 14)	PGY (n = 9)	
1	The game design is good	15(65.2)	10 (71.4)	5 (55.6)	0.367
2	Good quality of image, sound, and presenting	15 (65.2)	8 (57.1)	7 (77.8)	0.290
3	It’s good for preview	18 (78.3)	10 (71.4)	8 (88.9)	0.327
4	The test design is good for learning	19 (82.6)	12 (85.7)	7 (77.8)	0.517
5	It’s a good simulation game	16 (69.6)	9 (64.3)	7 (77.8)	0.418
6	The feedbacks are good for learning	15 (65.2)	8 (57.1)	7 (77.8)	0.290
7	The case scenarios are true to life	16 (69.6)	9 (64.3)	7 (77.8)	0.418

Fisher’s exact test.

### Qualification and academic achievements

Of the 34 (21 [61.8%] men; 13 [38.2%] women) dental students who participated in this study 22 (64.9%) reported that they often played video games. Regarding their learning strategies, ‘self-learning’ was rated as the most important strategy (n = 24, 70.6%), followed by ‘practice’ (n = 23, 67.6%), ‘reading’ (n = 20, 58.8%), ‘video’ (n = 19, 55.9%), ‘problem-based learning’ (n = 13, 38.2%), and ‘lectures’ (n = 11, 32.4%). When the aforementioned variables were compared to their qualification test scores and academic achievements in different specialties, there were no significant differences, except in the specialty of Oral Pathology & Maxillo-Facial Radiology for which men received higher average scores (Data not shown).

Regarding the VDC experience, 13 (38.2%) reported that they never used VDC as a self-learning tool during their clerkship, 9 (26.5%) reported that they used VDC once, 11 (32.4%) reported using VDC twice, and 1 (2.9%) using it thrice. Table 5 shows the associations between self-learning experience of VDC, the score on the qualification test, and academic achievements in different specialties. The results showed that the students who used VDC gained significantly higher scores on the qualification test. The VDC experiences may, thus, significantly predict higher scores on periodontics and endodontics.

**Table 5.** VDC experiences and student performances

Variables	Mean±standard deviation			p	LSD post comparison
	0^a^ (N = 13)	1^b^ (N = 9)	≥2^c^ (N = 12)		
**Qualification test**	70.00 ± 13.54	80.00 ± 5.59	79.58 ± 6.90	0.029*	b, c > a
**Academic achievements of clerkship**					
Oral Maxillofacial Surgery	84.58 ± 0.49	84.44 ± 1.13	84.42 ± 1.16	0.905	
Oral Pathology & Maxillo-Facial Radiology	78.80 ± 8.28	80.38 ± 5.64	80.14 ± 2.92	0.801	
Periodontics	82.46 ± 2.47	84.56 ± 2.19	82.33 ± 1.30	0.037*	b > a, c
Paediatric & Special Needs	85.11 ± 6.65	85.43 ± 5.89	88.49 ± 2.50	0.236	
Conservative Dentistry	84.92 ± 2.33	86.78 ± 1.48	86.42 ± 1.16	0.040*	b, c > a
Family Dentistry	85.69 ± 6.33	86.56 ± 1.24	86.50 ± 1.45	0.847	
Prosthodontics	77.85 ± 9.94	84.44 ± 2.35	82.17 ± 2.52	0.064	
Orthodontics	88.46 ± 2.70	89.89 ± 0.60	90.00 ± 0.85	0.074	
Overall score of clerkship	84.58 ± 0.49	84.44 ± 1.13	84.42 ± 1.16	0.905	

one-way ANOVA.

* p < 0.05.

### Practical application during the COVID-19 pandemic

There were 52 students who completed clerkship training in the Family Dentistry Department before the cautionary restrictions due to the pandemic from the Taiwan government, which began on 21 January 2020. Another 40 students finished their training during the pandemic itself. The number of patients reduced during this period and the students had more time to utilise the self-learning tools. In their homework, 18 (34.6%) students mentioned self-learning tools and only one mentioned VDC before the pandemic. After the outbreak of COVID-19 pandemic, 17 (42.5%) students reported the utilisation of self-learning tools (p = 0.440). However, a significantly higher proportion of students (n = 7, 17.5%) confirmed the value of VDC as an assistive tool for learning clinical reasoning (p = 0.019). The comments from students are summarised in Table 6. The students viewed the VDC as a self-directed learning tool which provided them with opportunities to practice treatment procedures and familiarise themselves with clinical operations, while simultaneously having fun.

**Table 6.** Summary of VDC mentioned in the students’ homework

No.	Quotes
S1	The learning resources for self-directed learning gave us an opportunity to practice, and the mini game on the computer further enhanced our understanding of treatment procedures and apparatus preparation.
S2	I really liked how there were suture practice kits and dentistry mini game in the clinic!
S3	Although sometimes there were few patients at the family dentistry clinic, this provided us with the opportunity to borrow the toolkit for self-directed learning and play interesting dentistry games. Thus, as a whole, I thoroughly enjoyed it!
S4	There were many tools and electronic mini games for clerks and interns to use for practice, and some dentists even took the initiative to teach us suture techniques. This gave us many opportunities for self-directed learning and hands-on operations, which was greatly beneficial to forming impressions in learning as well as knowledge retention.
S5	[In the clinic] there were some interactive learning resources (sutures, learning tools for anaesthetic injections, and clinic games), through which I could learn some basic knowledge while having fun at the same time.
S6	The self-directed learning area at the family dentistry clinical was cool. Answering questions in the mini games helped us familiarise ourselves with the necessary apparatus for different treatments. It was very practical.
S7	I think the learning environment at the family dental clinic was exceptional, and there were many learning resources for self-directed learning. This allowed us to practice some dental treatment procedures and methods in real life besides observing in the clinic. For example, there were learning equipment for OD fillings and scaling.
S8	With regard to the self-directed learning area, I am very thankful that the teachers provided us with opportunities to practice treatments using a simulated dental chair [VDC] so that we could familiarise ourselves with all the clinical operations.

## Discussion

This study describes the successful establishment of a simulated serious game for learning clinical reasoning in a dental clinic. The VDC showed good validity and a high potential for education in practice.

Using serious games can provide a safe and effective practice environment, but the development of these games must blend subject matter content, instructional design, learning objectives, and engaging game design to encourage learners to practice and develop their skills. The main goal of this study was to test VDC as an educational tool and to determine its effect on student knowledge and clinical reasoning; it may help close the gap between preclinical and clinical training. According to Bloom’s Taxonomy classification [22,23], there are six progressive levels of learning; from the foundation to the pinnacle, these are: knowledge, comprehension, application, analysis, synthesis, and evaluation. In this study, we assumed that the learner should be able to apply a concept, solve a problem by using or examining gained knowledge and understanding in some manner; hence, the game is targeted at the intermediate level in the Taxonomy. In the VDC game described above, the learner (player) must apply their knowledge of dental treatment procedures to answer questions, such as identifying the various instruments based on a given dental procedure. The game, hence, improves their clinical reasoning and learning outcome.

In this study, serious gaming successfully provided an alternate option for self-learning, and offered a similar experience and outcome to the other methods concerning dental education. The exploratory analysis showed that students who used VDC gained significantly higher scores on the qualification test and the VDC experiences significantly predicted better performances on periodontics and conservative dentistry. However, in the performance score of periodontics, the students who play VDC twice or more did not show a significant improvement. Periodontics requires more training in communication skills than conservative dentistry, to improve patients’ oral hygiene better. The VDC themes did not include scenes of virtual patients, which might be the cause of contradiction. In this study, at least the better knowledge gains and clinical reasoning skills in conservative dentistry can be attributed to playing VDC. In conservative dentistry, students learn to preserve teeth by treating caries and pulpitis. Students playing VDC showed an increase in their knowledge about treatment of caries and pulpitis. The results are similar to those of a previous study [24] in which students had a free choice on using a serious game in addition to formal teaching. Regardless of the type of study design, students using serious games appear to display at least similar, and sometimes even better, levels of knowledge and retention, skills development, and satisfaction than those not using these games [9,25,26].

Overall, the students and residents evaluated VDC as highly usable for preview and learning. Students’ and residents’ impression of VDC was positive. They had a higher positive impression of VDC in teaching clinical skills, effectiveness in education, and having fun. Playing with VDC was fun for the students, since they mentioned that they wanted to play again. This is an important precursor for motivated learning. Learners were motivated and reinforced to learn through fun and competition of serious games [27]. Although only 65.2% of the participants agreed that VDC is necessary for learning at the validation stage, it indicates a potential for application when real patients are not available during clinical practice.

Dental visits are a high-risk activity during the COVID-19 pandemic. After the COVID-19 outbreak, there was a reduction in the non-urgent dental care cases and dental emergency utilisation [28]. The impact of the pandemic has a certain influence on clinical skill training in clerkship which is the important infrastructure of dental education [1]. Based on the students’ feedback in this study, positive attitudes toward VDC use increased after the COVID-19 outbreak. It indicates that VDC provides an alternative way of learning for students during an extraordinary period when inflow of actual patients reduced. This further exhibits the usability of such games in teaching. On the other hand, serious games or virtual patients cannot replace the actual clinical training course because not all students may be interested in this learning style. Nevertheless, in the post-COVID-19 era, the model of dental education can be made more innovative to suit different situations and novel intelligent technology should be applied for future dental education [1].

### Limitations

This study had some limitations. Firstly, the case scenarios were limited to tooth caries and tooth pulpitis, and the procedural knowledge was assessed concerning only these clinical conditions. Thus, future studies should include a wider range of clinical cases. Secondly, the game quality of VDC was acceptable but not optimal; its user friendliness should be enhanced. Thirdly, the data could not be collected using the logging system of the game; this included when and how long each student logged into the game, how many times each student submitted their answers, and what answers they submitted. The future VDC should rectify these limitations to achieve a better serious game.

Finally, in this study, students’ game-playing habit and learning strategies were not found to have a significant influence on their academic performance; however, there may be other variables that confound the results. For example, academic performance before clerkship was not considered in this study. Considering the relearning feature of game design and the lack of a function for data collection, convenience sampling and quasi-experimental ex post facto design were adopted in this study’s validation process. Therefore, it is suggested that further research use rigorous randomised control trials to confirm/support this study’s findings.

### Conclusions

The VDC as an educational tool and its effectiveness on clinical reasoning skill and knowledge gain among clerkship dental students have been validated and confirmed in this study. Students using VDC gain significantly higher scores on performance and qualification test. In addition, the VDC shows a high potential as an assistive self-learning tool in clinical training environment, especially relevant and pertinent after the COVID-19 outbreak.

## Appendix. The validation procedure of the content validity

**Table:**

Themes	Original items	Average score	ICVI	Failed	Modified items	Average score	ICVI
(1) Dental clinic environment	1. Where is the basic setup?	4.6	1				
	2. Where is the alginate mixer?	3.4	0.4	V	Where is the upper stock tray (No. 12)?	4.4	1
	3. Where is topical anaesthetic agent?	4.2	0.6	V	Where is the local anaesthetic cartridge (no epinephrine solution)?	4.4	1
	4. Where is the surgical mask?	4.6	1				
	5. Where is the dental health history form?	4.8	1				
	6. Where is the light curing machine?	4.6	1				
	7. Where is the apex locator?	4.8	1				
	8. Where is the automated electronic blood pressure device?	4.8	1				
	9. Where is the disclosing agent?	4.8	1				
	10. Where is the articulating paper?	4.4	1				
(2) Dental material	1. Which is the correct mixing zinc oxide-eugenol for the temporary cementation?	4.4	0.8				
	2. Which is the correct mixing zinc phosphate for the permanent cement?	4.8	1				
(3) Whole-person dental care: first visit (OD)	1. Which are personal protective equipment before the treatment?	3.6	0.6	V	What do you need to wear before the treatment?	4.4	0.8
	2. Which are the basic instruments that are needed before the oral examination of Miss Luo?	3.8	0.6	V	Which are the basic instruments that are needed from the dental unit before the oral examination of Miss Luo?	4.8	1
	3. According to treatment plan for tooth filling, which equipment and supplies are needed for cavity preparation?	3.8	0.8				
	4. Miss Luo chokes on water during the tooth cavity preparation. Which items did you fail to prepare?	4.2	1				
	5. Which items do you need to prepare for the composite resin filling for tooth cavities?	3.6	0.8				
	6. Which items do you need to adjust after filling the tooth cavity, besides the items that have been placed?	3.4	0.6	V	Which items that are absent from the dental unit do you need to adjust to fill the tooth cavity?	4.4	1
(4) Whole-person dental care: second visit (ENDO)	1. Which document and equipment do you need to prepare for Miss Luo to accept topical anaesthetic before the root canal treatment?	3.6	0.6	V	Which document and inspection do you need to prepare before Miss Luo accepts the topical anaesthetic?	4.6	1
	2. Which items do you need to prepare for Miss Luo to accept the topical anaesthetic before the root canal treatment?	4.0	0.8				
	3. Which items do you need to prepare and place for the rubber dam besides the basic setup?	4.0	0.8				
	4. Which items are needed to open the chamber besides the aforementioned equipment, supplies, and X-ray?	4.4	1				
	5. Which items are need for the root canal enlargement procedure?	4.0	0.8				
	6. Which items are needed for the root canal filling procedure?	3.6	0.8				
(5) X-ray arrangement	1. Upper right second molar	4.4	1				
	2. Upper right second premolar	4.4	1				
	3. Upper right canine	4.6	1				
	4. Upper right central incisor	4.6	1				
	5. Upper left canine	4.6	1				
	6. Upper left second premolar	4.4	1				
	7. Upper left second molar	4.2	0.8				
	8. Lower left second molar	4.4	1				
	9. Lower left central incisor	4.4	1				
	10. Lower right canine	4.6	1				
(6) Orthodontics	1. Which instrument or method can be used to assist with the elastomeric ring separators placement before the orthodontic treatment?	4.6	1				
	2. Which are the three items that can be used to assist with the orthodontic bands’ placement?	4.6	1				
	3. Which item is required to hold the bracket?	4.8	1				
	4. Which are the items and tools that can be used to assist with the ligature ties’ placement?	4.2	1				
	5. Which items can be used to remove the orthodontic bands?	4.6	1				
	6. Which item that has a smooth and rounded head can be used to remove and bend orthodontic wires?	4.6	1				
	7. Which are the items that can be used to remove and place elastomeric ties?	4.2	1				
	8. Which are the five basic kits for orthodontic treatment in the KMUH?	4.4	1				

S-CVI/UA = 34/42 = 0.81.

S-CVI/Ave = (8 × 0.8 + 34)/42 = 0.96.
